# The Effect of Pembrolizumab in Absence of Programmed Death 1 Receptor

**DOI:** 10.7759/cureus.2896

**Published:** 2018-06-29

**Authors:** Layth Al Attar, Phu Truong

**Affiliations:** 1 Internal Medicine, University of Kansas School of Medicine, Kansas City, USA; 2 Cancer Center of Kansas, University of Kansas School of Medicine, Kansas City, USA

**Keywords:** pdl-1, immunotherapy, pd-1 inhibitor, solid tumor

## Abstract

Tumor therapy has evolved greatly since the discovery of immunotherapies. New therapies permit targeted approach with less side effects. Immunotherapies target specific components recognized on tumor cells, such as Programmed death-1 (PD-1). Overexpression of PD-1 markers in solid gastrointestinal tumors is a consequence of deficiency in DNA mismatch repair mechanism. Pembrolizumab is an example of immunotherapy targeting PD-1. Pembrolizumab binding of PD-1 helps the immune system recognize tumor cells and initiate destruction cascade. We present a case of a PD-1 negative appendiceal tumor responding to pembrolizumab.

## Introduction

Cancer therapy has evolved greatly in recent years. It has seen a shift towards cell-marker therapy agents. This is contributed to the significant discoveries in fields of immunology and oncology. Development of immunotherapies opened the doors to dispute the deadliest characteristic of cancer cells. Immunotherapies hinder the ability of cancer cells to evade the immune system, allowing them to grow and spread. Advancements in monoclonal antibodies have shown unprecedented rates of durable clinical response in patients with various cancer types.

Tumors evade immune destruction by one of the following mechanisms: alteration of antigenic expression that permits T-cell recognition [1], promotes an immune-tolerant microenvironment [2], or upregulates the expression of immune checkpoint molecules such as programmed death-1 ligand (PD-L1) [3]. Programmed death-1 (PD-1)/PD-L1 have allowed for the development of monoclonal antibodies specific to PD-1/PD-L1 to combat tumors.

Solid tumors have been shown to benefit from administration of immunotherapy [4]. This is related to deficiency in DNA mismatch repair (dMMR), rendering tumors to harbor thousands of mutations. In such situation, tumors are prone to harbor thousands of PD-L1 on their surface [5]. These tumors with MMR are highly sensitive to PD-1 blockade therapy [5]. We present a case of metastatic signet goblet cell carcinoma of the appendix that responded to immunotherapy, although there was no evidence of mismatch repair defect.

## Case presentation

A 63-year-old female presented in 2012 for increased abdominal girth. It was associated with nausea, vomiting, constipation, and unintentional weight loss of 10 pounds. Initial abdomen-pelvic computed tomography (CT) scan showed a very large mixed cystic and solid mass process arising from the pelvis into the abdomen of suspected ovarian origin. The patient was diagnosed with primary appendiceal adenocarcinoma based on pathology evaluation obtained after total abdominal hysterectomy with bilateral salpingo-oophorectomy, omentectomy, and right hemicolectomy with appendectomy. At this point, she had evidence of stage IV appendiceal adenocarcinoma.

The patient was started on a chemotherapy regimen consisting of folinic acid, fluorouracil, and oxaliplatin for six months. After three years, follow-up CT-scan surveillance confirmed progression of retroperitoneal lymphadenopathy. A second line chemotherapy regimen of folinic acid, fluorouracil, and irinotecan plus avastin was started. She had eight courses and CT imaging showed improvement in the left retroperitoneal lymphadenopathy. Unfortunately, she developed hypertension, deep vein thrombosis, and significant nausea. These were side effects related to her chemotherapy regimen. She was given treatment holiday. On return visit, CT-scan showed advancement and burden of metastatic disease with hepatic lesions. Additionally, carcinoembryonic antigen (CEA) was elevated. She was started on a second round of folinic acid, dose reduced fluorouracil, and oxaliplatin.

While on chemotherapy, she developed new left supraclavicular lymph node, and CEA continued to increase. Genetic testing revealed a negative result for KRAS of wild-type, absence of microsatellite instability, and negative PDL-1 testing. With these findings, the patient’s medications were switched to irinotecan and vectibix. Clinical improvement started to be noticed, but the patient acquired a hypersensitivity reaction contributed to vectibix.

**Figure 1:**
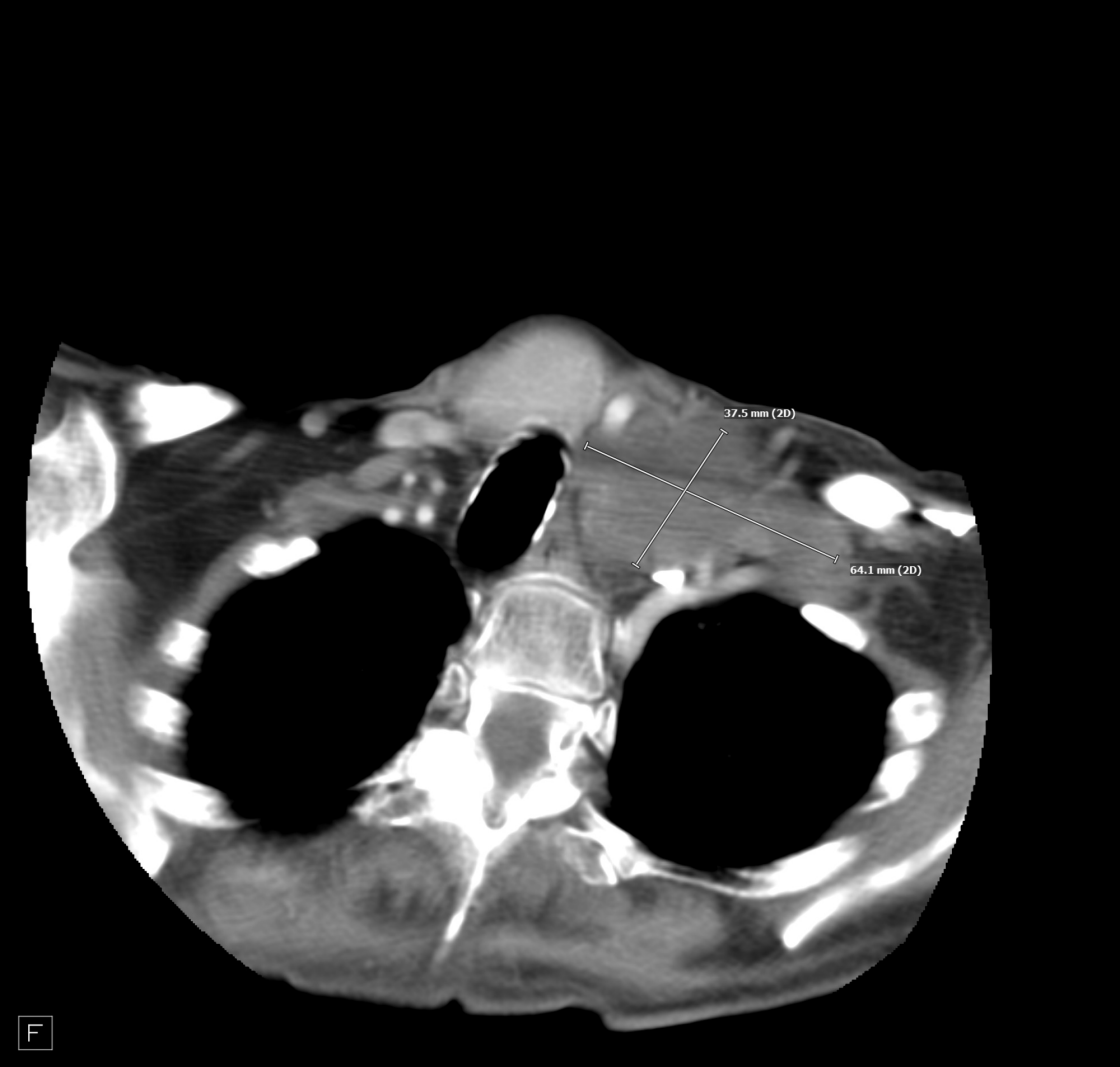
Left supraclavicular adenopathy.

**Figure 2:**
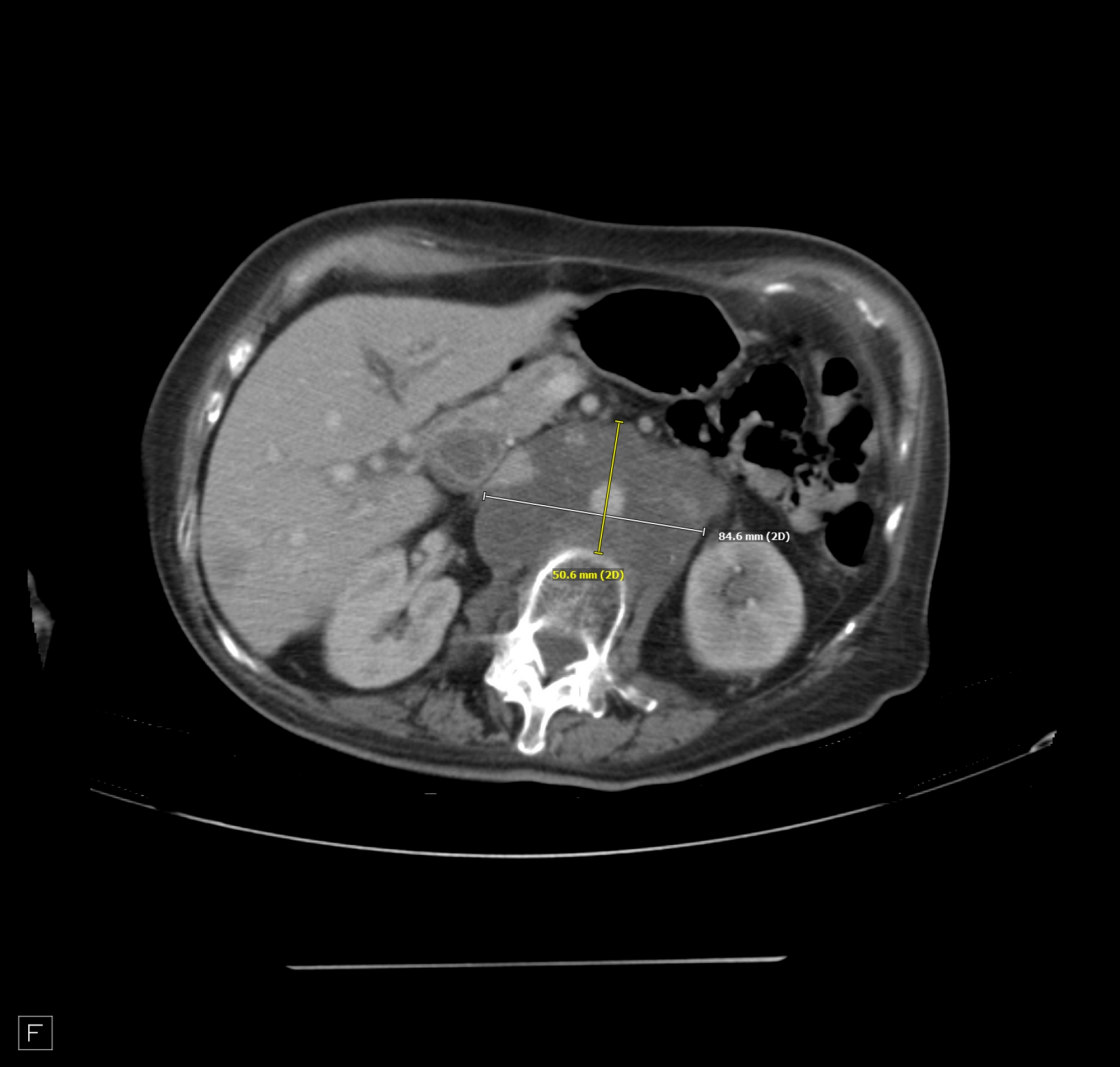
Retroperitoneal adenopathy encasing the aorta and partially encasing inferior vena cava.

**Figure 3:**
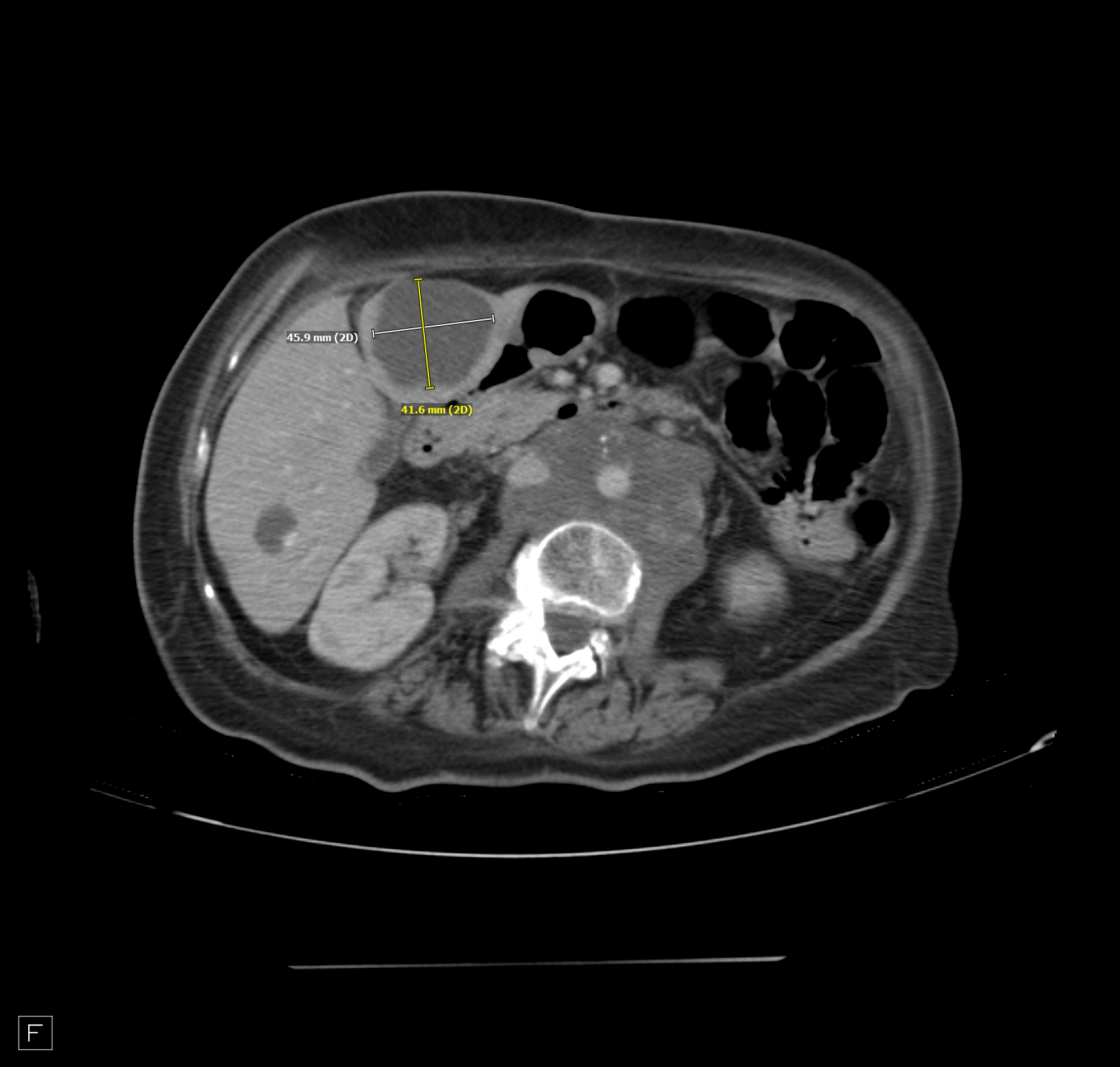
Hepatic metastatic disease with slight decreased size.

**Figure 4:**
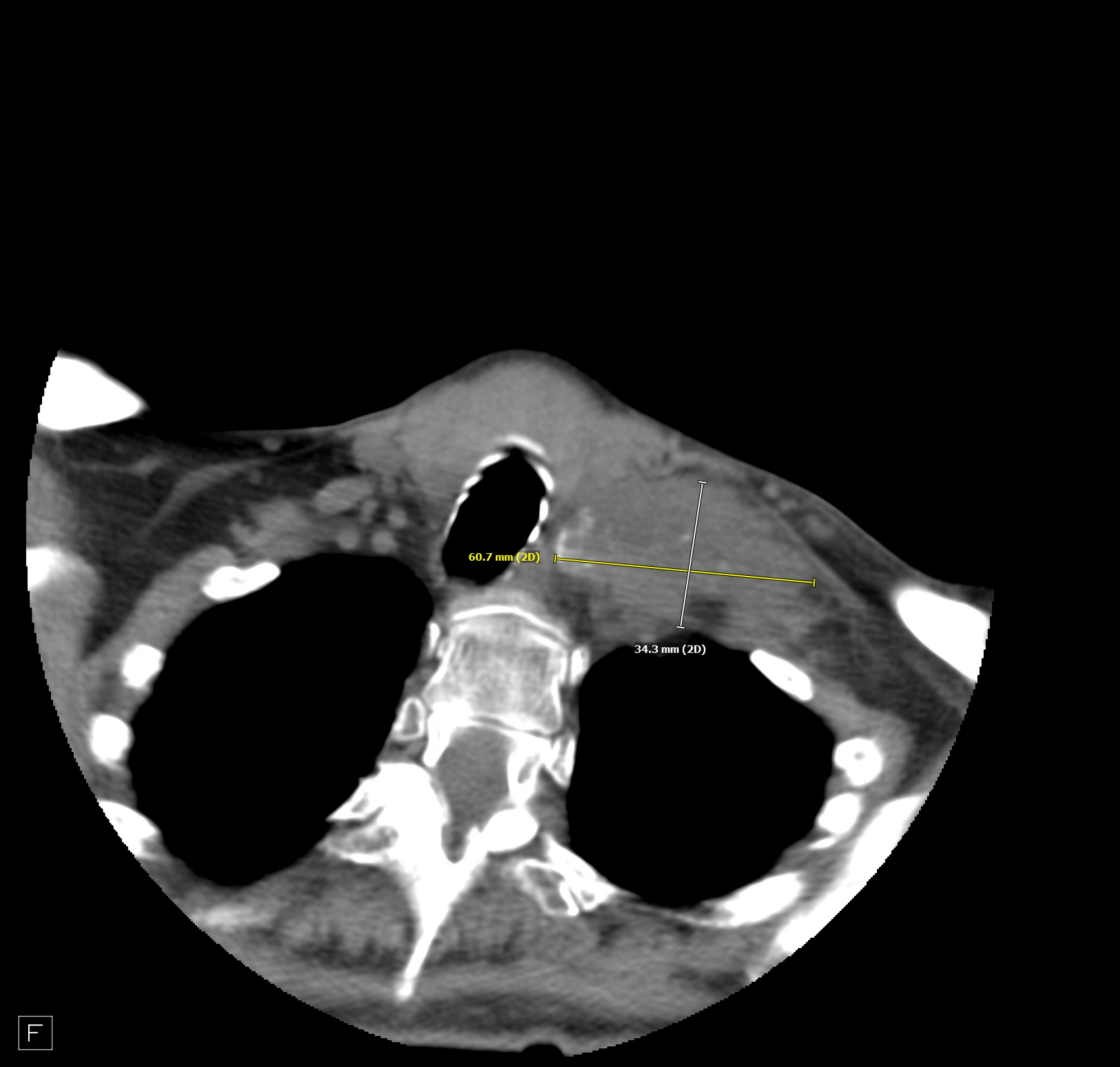
Left supraclavicular adenopathy with calcification of the conglomerate.

**Figure 5:**
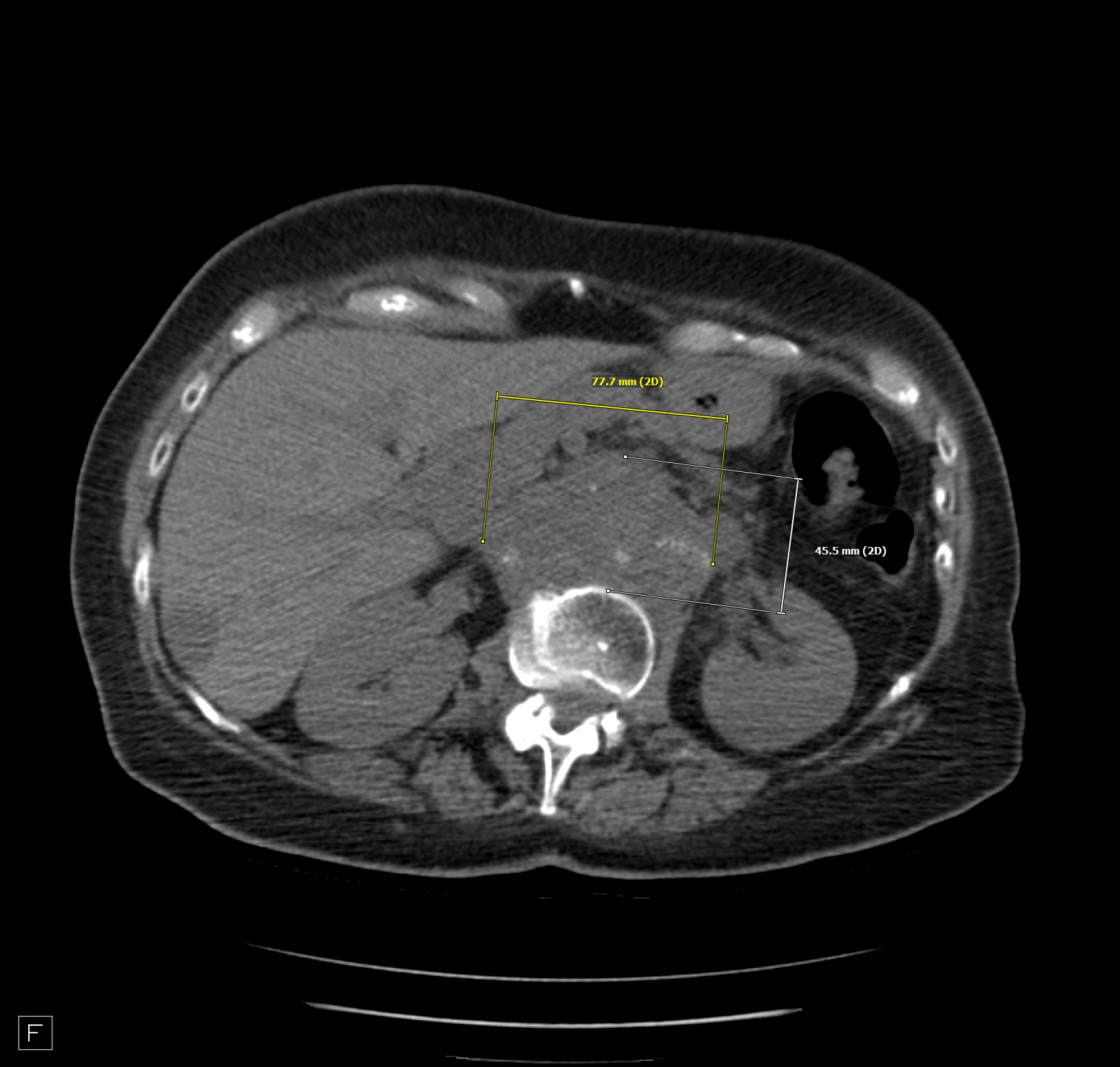
Retroperitoneal adenopathy encasing the aorta and inferior vena cava.

The patient insisted on continuing treatment. She was started on pembrolizumab, although tests did not show microsatellite instability. After the first dose of pembrolizumab, she reported resolution of her abdominal pain and experiencing regular bowel movements. CEA was reported 95 ng/mL on a test done 10 days after the first dose. The test was repeated monthly to monitor pembrolizumab benefit. Results showed a downwards trend to 80 then 43 ng/mL. CT scan was repeated two months after initiation of pembrolizumab. It showed an increase from previous left supraclavicular adenopathy (Figure 1), stable retroperitoneal periaortic lymphadenopathy (Figure 2), and decreased size of hepatic metastasis (Figure 3). The patient continued on this medication with notable clinical improvement. The patient completed 10 doses over a period of six months. A repeat CT scan showed increased calcification in relatively similar left supraclavicular lymphadenopathy (Figure 4) and retroperitoneal adenopathy (Figure 5). Hepatic metastasis lesion was similar in size from previous scans. A more recent CEA test showed an astonishing result, with CEA dropping to 1.9 ng/mL.

## Discussion

Our case failed recommended chemotherapies for primary appendiceal adenocarcinoma. Also, the recommended chemotherapy for primary appendicular carcinoma is associated with significant side effects [6]. These side effects have been devastating to many patients to the extent of stopping therapy. The pursuit of high selectivity allowed for the discovery of immunotherapy. Selectivity is achieved by utilization of the human immune system to attack tumor cells by stimulating a specific cell line. Immunotherapy has different modalities such as monoclonal antibodies recognizing specific cancer cell parts, immune checkpoint inhibitors, or cancer vaccines.

In normal conditions, the PD-1/PD-L1 pathway downregulates the immune response [4]. Activation of this immune checkpoint inhibitor pathway, by tumor cells, allows for their evasion of an antigen-specific T-cell immunologic response [6,7]. Overexpression of PD-L1 serves as a protective mechanism of tumor cells. This overexpression has been correlated with deficiency in DNA mismatch repair [3,4,8,9]. Kim et al. [8] examined different anatomic tumor types. They identified PD-L1 expression in 38.9% of the dMMR tumors, 15.2% of MMR proficient (pMMR) tumors, and 2.8% in tumors with unknown MMR status [8]. A recent series of colorectal cancer (CRC) evaluation to assess PD-L1 by immunohistochemistry suggests increased PD-L1+ in dMMR tumors with 18% dMMR CRC staining positive versus 2% of pMMR CRC [10]. Mismatch repair is a cellular defense mechanism that recognizes and repairs mismatch of bases during DNA replication and recombination [11]. In humans, MMR proteins of MLH1, MLH3, MSH2, MSH3, MSH6, PMS1, and PMS2 demonstrate this DNA homeostasis [12]. These proteins lose their function by genetic alteration with elevated rates of base substitution and frameshift mutations, or missense mutation that yields a stable yet functionally inactive MMR protein. This state of genetic hypermutability secondary to dMMR is referred to by microsatellite instability (MSI). MSI can be hereditary or sporadic [13]. Lynch syndrome is an example of hereditary pattern. Sporadic dMMR mutation is rare, yet epigenetic silencing is more likely to reduce MMR gene expression [13]. Approximately 15% of spontaneous colorectal cancers are MSI positive. The majority of these are caused by epigenetic silencing of MLH1 [13]. A potential benefit in POLE gene mutation and tumor mutational burden (TMB) exists to determine candidacy for immune checkpoint inhibitor therapy [8,14,15]. Both POLE and TMB are suggested additional tests to MSI testing aiming to improve patient selection.

## Conclusions

In conclusion, there has been remarkable response to PD-1 inhibitors in mismatch repair deficient gastrointestinal tumors. To the best of our knowledge, this is the first report of a gastrointestinal tumor that is PDL-1 negative and without microsatellite instability showing impressive response to pembrolizumab. This case revealed a potential benefit of pembrolizumab without diagnostic evidence of MSI or presence of PDL-1.

## References

[1] Donia M, Andersen R, Kjeldsen JW (2015). Expression of MHC class II in melanoma attracts inflammatory tumor-specific CD4+ T- cells, which dampen CD8+ T-cell antitumor reactivity. Cancer Res.

[2] Guo F, Wang Y, Liu J, Mok SC, Xue F, Zhang W (2016). CXCL12/CXCR4: a symbiotic bridge linking cancer cells and their stromal neighbors in oncogenic communication networks. Oncogene.

[3] Tumeh PC, Harview CL, Yearley JH (2014). PD-1 blockade induces responses by inhibiting adaptive immune resistance. Nature.

[4] Gong J, Chehrazi-Raffle A, Reddi S, Salgia R (2018). Development of PD-1 and PD-L1 inhibitors as a form of cancer immunotherapy: a comprehensive review of registration trials and future considerations. J Immunother Cancer.

[5] Lee V, Murphy A, Le DT, Diaz LA Jr (2016). Mismatch repair deficiency and response to immune checkpoint blockade. Oncologist.

[6] Liang XJ, Chen C, Zhao Y, Wang PC (2010). Circumventing tumor resistance to chemotherapy by nanotechnology. Methods Mol Biol.

[7] Topalian SL, Drake CG, Pardoll DM (2012). Targeting the PD-1/B7-H1(PD-L1) pathway to activate anti-tumor immunity. Curr Opin Immunol.

[8] Kim ST, Klempner SJ, Park SH (2017). Correlating programmed death ligand 1 (PD-L1) expression, mismatch repair deficiency, and outcomes across tumor types: implications for immunotherapy. Oncotarget.

[9] Sloan EA, Ring KL, Willis BC, Modesitt SC, Mills AM (2018). PD-L1 expression in mismatch repair-deficient endometrial carcinomas, including lynch syndrome-associated and MLH1 promoter hypermethylated tumors. Am J Surg Pathol.

[10] Lee LH, Cavalcanti MS, Segal NH (2016). Patterns and prognostic relevance of PD-1 and PD-L1 expression in colorectal carcinoma. Mod Pathol.

[11] Iyer RR, Pluciennik A, Burdett V, Modrich PL (2006). DNA mismatch repair: functions and mechanisms. Chem Rev.

[12] Jascur T, Boland CR (2006). Structure and function of the components of the human DNA mismatch repair system. Int J Cancer.

[13] Jenkins MA, Hayashi S, O'Shea AM (2007). Pathology features in Bethesda guidelines predict colorectal cancer microsatellite instability: a population-based study. Gastroenterology.

[14] Mehnert JM, Panda A, Zhong H (2016). Immune activation and response to pembrolizumab in POLE-mutant endometrial cancer. J Clin Invest.

[15] Gong J, Wang C, Lee PP, Chu P, Fakih M (2017). Response to PD-1 blockade in microsatellite stable metastatic colorectal cancer harboring a POLE mutation. J Natl Compr Canc Netw.

